# Menin inhibitors for acute myeloid leukemia: latest updates from the 2023 ASH Annual Meeting

**DOI:** 10.1186/s13045-024-01573-2

**Published:** 2024-07-19

**Authors:** Zhuo-Yu An, Xiao-Hui Zhang

**Affiliations:** 1 Peking University People’s Hospital, Peking University Institute of Hematology, Beijing, China; 2 Collaborative Innovation Center of Hematology, Peking University, Beijing, China; 3 Beijing Key Laboratory of Hematopoietic Stem Cell Transplantation, Beijing, China; 4 National Clinical Research Center for Hematologic Disease, Beijing, China

**Keywords:** Menin inhibitors, Acute myeloid leukemia, Revumenib, Ziftomenib, Clinical trials

## Abstract

Recent developments in menin inhibitors for relapsed or refractory acute myeloid leukemia (AML) were highlighted at the 2023 ASH Annual Meeting. Notably, revumenib showed promising efficacy, achieving a 100% ORR when combined with decitabine/cedazuridine and venetoclax. These findings underscore the potential of menin inhibitors in transforming AML treatment, particularly in genetically defined subgroups, offering hope for improved patient outcomes. Ongoing studies, like KOMET-008, further explore the synergistic potential of menin inhibitors in combination regimens, shaping future AML management strategies.

## To the editor

Acute myeloid leukemia (AML) remains challenging, particularly subtypes characterized by genomic instability, such as KMT2A rearrangement and NPM1 mutations [1, 2]. Current treatment regimens, including chemotherapy and targeted therapies, often lead to limited long-term success and significant toxicity. Menin inhibitors have emerged as a promising therapeutic approach, particularly for AML subtypes with specific genetic abnormalities such as KMT2A rearrangements and NPM1 mutations (Table 1). Here we synthesize findings from the 2023 ASH Annual Meeting, focusing on the efficacy and safety of Menin inhibitors across various clinical settings.

**Table 1:**
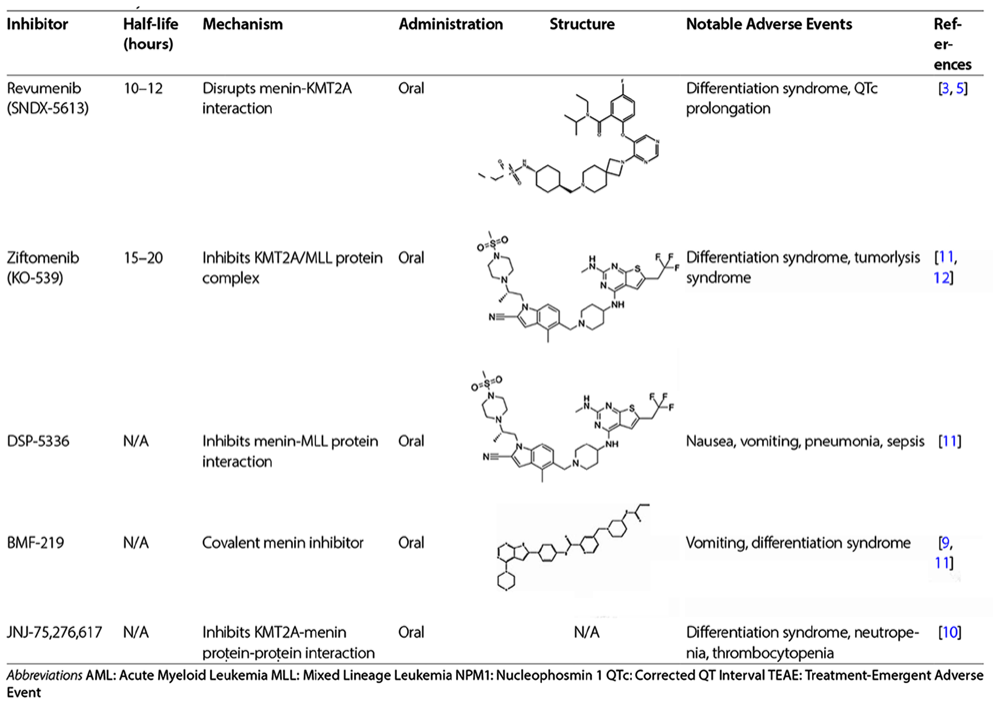
Summary of menin inhibitors

## Revumenib (SNDX-5613)

Revumenib, a selective Menin inhibitor, has shown substantial efficacy, particularly for AML patients with KMT2A rearrangements (KMT2Ar). Issa et al. reported significant outcomes in a Phase I/II trial where 7 evaluable patients with relapsed or refractory(r/r) AML characterized by KMT2A rearrangements were assessed. Revumenib combined with decitabine/cedazuridine and venetoclax achieved a 100% overall response rate (ORR), with all evaluated patients attaining morphological remission [3].

Further, Zucenka et al. discussed the outcomes of Revumenib maintenance therapy post-hematopoietic stem cell transplant (HSCT) in the AUGMENT-101 study. Of 131 treated patients, nine resumed Revumenib post-transplant, with treatment durations ranging from 23 to 588 days. Notably, six of the nine patients who resumed Revumenib post-transplant maintained complete response (CR), and five achieved or sustained measurable residual disease (MRD) negativity [4].

Additionally, pivotal Phase 2 results from AUGMENT-101, as reported by Aldoss et al., confirmed Revumenib’s robust clinical profile. Among 94 treated patients with relapsed/refractory KMT2Ar acute leukemia, the CR + CR with partial hematologic recovery (CRh) rate was 22.8%, the composite complete response rate (CRc) was 43.9%, and the ORR was 63.2% [5].

Revumenib’s safety profile was manageable, with differentiation syndrome and QTc prolongation [3–6] (Table 2).

## Ziftomenib

Ziftomenib, a menin-KMT2A interaction inhibitor, targets NPM1-mutated and KMT2A-rearranged AML. The ongoing phase 1 KOMET-008 trial is investigating the agent plus gilteritinib, FLAG-IDA (fludarabine, cytarabine, granulocyte-colony stimulating factor, and idarubicin), or low-dose cytarabine in patients with r/r NPM1-mutant or KMT2A-rearranged AML [7]. Komet-001 results indicated significant clinical activity, with a 40% complete remission rate and a 45% ORR in 20 patients [8]. These promising outcomes led the FDA to grant breakthrough therapy designation for Ziftomenib in NPM1-mutated AML (Table 2).

**Table 2:** Key clinical outcomes of Menin inhibitors in ASH 2023

Inhibitor	Study Name	Patient Population	Key Efficacy Outcomes	Safety Profile
Revumenib	AUGMENT-101	Relapsed/refractory KMT2Ar AML	CR + CRh: 22.8% (21/94), CRc: 43.9% (41/94), ORR: 63.2% (59/94)	Manageable; differentiation syndrome (16.0%), febrile neutropenia (13.8%), and QTc prolongation (13.8%);
Revumenib	Post-HSCT Study	Post-HSCT patients	CR: 6/9, MRD negativity: 5/9	Manageable; no new safety signals, common AEs included cytopenias (20%) and infections (10%)
Ziftomenib	Komet-008	Relapsed/refractory AML	Not available	Manageable; common AEs included anemia (25%), pneumonia (20%), thrombocytopenia (15%), neutropenia (15%)
DSP-5336	N/A	MLLr and NPM1 mutated AML	CR with incomplete recovery: 1/6, morphologic leukemia-free state: 1/6	Not available
BMF-219	COVALENT-101	Relapsed/refractory acute leukemia	CR: 2/5	Vomiting 13% (3) and Differentiation Syndrome (DS) 13% (3). No Grade 5 TRAEs were reported.
JNJ-75,276,617	Phase 1 Study	Relapsed/refractory AML	CR + CRh: 22.8% (21/94), CRc: 43.9% (41/94), ORR: 63.2% (59/94)	Differentiation syndrome (8 [14%]), neutropenia (6 [10%]), anemia and thrombocytopenia (4 [7%] each).

*Abbreviations* AE: Adverse Event AML: Acute Myeloid Leukemia CR: Complete Remission CRh: Complete Remission with Partial Hematologic Recovery CRc: Composite Complete Remission HSCT: Hematopoietic Stem Cell Transplant KMT2Ar: KMT2A rearrangements MLL: Mixed Lineage Leukemia MRD: Measurable Residual Disease NPM1: Nucleophosmin 1 ORR: Overall Response Rate QTc: Corrected QT Interval TEAE: Treatment-Emergent Adverse Event

## DSP-5336

DSP-5336, an investigational oral menin inhibitor, targets the menin and MLL protein interaction. The dose escalation portion of the DSP-5336 study consists of two parallel arms (Arm A: without concomitant anti-fungal azole therapy; Arm B: with concomitant azole therapy). Among six MLLr patients treated, one achieved a five-month CR, another maintained a morphologic leukemia-free state, and one had stable disease. For four NPM1 patients, treatment resulted in stable disease [9] (Table 2).

### BMF-219

BMF-219, a pioneering covalent menin inhibitor, is under evaluation in the COVALENT-101 study (NCT05153330), which included adults with r/r acute leukemia ineligible for standard therapy. Among the efficacy evaluable population, two out of five patients achieved complete remission [10] (Table 2).

### JNJ-75,276,617

In the Phase 1 study of JNJ-75,276,617(NCT04811560), conducted by Jabbour et al., this menin-KMT2A inhibitor was evaluated in adult patients with r/r AML harboring KMT2A or NPM1 mutations. From the 58 patients enrolled, 97% had r/r AML, and 3% had ALL, adverse events were manageable(Table 2). The clinical efficacy was highlighted by a 63% reduction in bone marrow disease burden. At the highest dose tested, the ORR was 50%, the time to response ranged from 1.0 to 3.3 months [11] (Table 2).

### Safety and adverse events

The safety profile of menin inhibitors was manageable. Common adverse events included gastrointestinal symptoms, cytopenias, and, less frequently, differentiation syndrome, which were generally reversible with dose adjustments or supportive care [6] (Table 2).

Traditional AML treatments like intensive chemotherapy and HSCT often lead to significant toxicities and limited efficacy, especially in relapsed/refractory cases. Menin inhibitors have shown in clinical trials to induce remission in heavily pretreated populations with a more manageable safety profile. They present fewer severe adverse events and effective management of differentiation syndrome, making them a significant advancement in AML management.

The 2023 ASH Annual Meeting highlighted the transformative potential of menin inhibitors, especially for genetically defined subgroups. Future research should explore their synergistic potential with other therapeutic agents, such as hypomethylating agents and BCL-2 inhibitors, to enhance treatment efficacy and assess long-term response durability.

## Data Availability

No datasets were generated or analysed during the current study.

## Abbreviations

AML: Acute Myeloid Leukemia
CR: Complete Remission
CRh: Complete Remission with Partial Hematologic Recovery
CRc: Composite Complete Remission
ORR: Overall Response Rate
HSCT: Hematopoietic Stem Cell Transplant
MRD: Measurable Residual Disease
